# The role of household food insecurity in malnutrition among Indonesian children under 5 years of age: a systematic review and meta-analysis (2015–2025)

**DOI:** 10.1017/S1368980026102365

**Published:** 2026-03-26

**Authors:** Rahayu Sutrisno, Irka Dwi Fatmawati, Agung Dwi Laksono, Widya Rahmawati, Nia Novita Wirawan, Dian Handayani

**Affiliations:** 1 Master of Nutrition Science Study Program, Faculty of Health Sciences, Universitas Brawijaya, Indonesia; 2 Research Center of Public Health and Nutrition, National Research and Innovation Agency Republic of Indonesia, Indonesia; 3 Nutrition Department, Faculty of Health Sciences, Universitas Brawijaya, Indonesia

**Keywords:** Child malnutrition, Household food insecurity, Indonesia, Meta-analysis, Systematic review

## Abstract

**Objective:**

To synthesise and quantify the association between household food insecurity (HFI) and various forms of malnutrition that include stunting, wasting, underweight, overnutrition and anaemia among Indonesian children under 5 years of age.

**Design:**

A systematic review and meta-analysis was conducted in accordance with Preferred Reporting Items for Systematic Reviews and Meta-Analyses (PRISMA) 2020 guidelines. The study included literature search, screening, data extraction, quality assessment using Joanna Briggs Institute (JBI) tools and meta-analysis using Review Manager 5.4.

**Setting:**

Studies conducted in Indonesia, covering urban, rural and mixed settings across multiple provinces.

**Participants:**

Children under 5 years of age residing in Indonesia, from households assessed for food insecurity using validated tools.

**Results:**

A total of thirty-two studies met the inclusion criteria, of which twenty-six were eligible for meta-analysis. HFI was significantly associated with higher odds of stunting (case–control: OR = 4·66; 95 % CI: 3·39, 6·40; *P* < 0·001; cross-sectional: OR = 4·61; 95 % CI: 4·17, 5·11; *P* < 0·001), wasting (OR = 1·92; 95 % CI: 1·60, 2·32; *P* < 0·001), underweight (OR = 5·26; 95 % CI: 2·12, 13·04; *P* < 0·001) and overnutrition (OR = 1·66; 95 % CI: 1·49, 1·85; *P* < 0·001). Children in food-secure households had significantly lower odds of anaemia (OR = 0·41; 95 % CI: 0·30, 0·58; *P* < 0·001).

**Conclusions:**

HFI is strongly associated with multiple forms of malnutrition among Indonesian children under 5 years of age. These findings highlight the urgent need for integrated, nutrition-sensitive strategies that address food security to improve child health and reduce malnutrition in Indonesia.

Malnutrition among children under 5 years of age remains a persistent global health challenge, disproportionately affecting low- and middle-income countries^(1,2)^. Indonesia exemplifies this burden, where despite steady economic progress, the prevalence of multiple forms of malnutrition, including stunting, wasting, underweight, overweight and anaemia, remains unacceptably high. According to the 2023 Indonesia Health Survey, 21·5 % of children under 5 years of age were stunted, 15·9 % were underweight, 8·5 % experienced wasting, 4·2 % were classified as overweight and 23·8 % suffered from Fe deficiency anaemia^(3)^. These figures reflect a persistent nutritional problem and show the complexity of nutritional issues among young children.

The consequences of malnutrition in early childhood are both profound and far-reaching. Stunting and wasting are associated with impaired cognitive development, diminished physical growth and decreased educational attainment and productivity in adulthood^(4)^. Fe deficiency anaemia further exacerbates developmental delays, increases the incidence of infection and reduces academic performance and long-term earning potential^(5–7)^. Beyond individual health impacts, the economic consequences of malnutrition are considerable. Undernutrition has been estimated to reduce gross domestic product by up to 3·0 % in affected countries due to increased healthcare costs and reduced workforce productivity^(8)^. Conversely, childhood overweight contributes to rising healthcare expenditures, with annual medical costs increasing by as much as USD 237·6 per capita^(9)^. It means that child malnutrition is not only a health crisis but also a critical barrier to sustainable development.

In response to the persistent burden of child malnutrition, the Government of Indonesia has implemented multisectoral interventions through national strategies such as the Stunting Reduction Acceleration Program and the Family Hope Program (*Program Keluarga Harapan*/PKH), which target early childhood care, maternal nutrition and household economic support. These programmes aim to reduce food insecurity and improve child nutrition outcomes through both nutrition-specific and nutrition-sensitive approaches^(10–12)^. However, the effectiveness of such interventions remains contingent on a deeper understanding of proximal determinants like household food security (HFS).

At the centre of these interventions lies the concept of HFS, defined by the FAO as consistent physical, social and economic access to sufficient, safe and nutritious food for all individuals^(13)^. The World Food Programme expands this framework into three interdependent dimensions: food availability, access and utilisation. United States Agency for International Development (USAID) adds further emphasis on food system resilience and dietary quality as essential components^(13,14)^. In the context of child nutrition, HFS functions as a proximal determinant, directly influencing dietary intake, feeding behaviour and health service utilisation^(15)^.

Despite the well-established pathways, previous research reveals inconsistencies in the association between household food insecurity (HFI) and child malnutrition^(16,17)^. Systematic reviews confirm the correlation between food insecurity and stunting, underweight and wasting, yet note variations in strength and direction across contexts^(15,18)^. Many observational studies report increased risk of poor nutritional outcomes among food-insecure households, including the dual burden of stunting and overweight^(12,19)^. However, these findings are often derived from heterogeneous regions, limiting their applicability to Indonesia. The unique sociocultural, economic and geographical landscape of Indonesia necessitates context-specific analysis.

This study aims to synthesise and quantify the association between HFI and five forms of malnutrition, namely stunting, underweight, wasting, overnutrition and anaemia, among children under 5 years of age in Indonesia using systematic review and meta-analytic methods. By encompassing this range of nutritional outcomes, the review intends to capture the full spectrum of health risks associated with food insecurity in early childhood. This approach facilitates the identification of critical intervention points and the development of targeted, evidence-informed recommendations. Ultimately, the study aims not only to synthesise current scientific knowledge but also to provide evidence that can support the development of sustainable, context-sensitive nutrition strategies to address the diverse and persistent nutritional challenges across Indonesia.

## Methods

This systematic review was prospectively registered with the International Prospective Register of Systematic Reviews (PROSPERO) under registration number CRD420251013791. The review was conducted in accordance with the Preferred Reporting Items for Systematic Reviews and Meta-Analyses (PRISMA) 2020 guidelines to ensure methodological rigour, transparency and reproducibility^(20)^. The review was designed to answer the following research question: ‘What is the association between HFS and multiple forms of malnutrition, including stunting, underweight, wasting, overweight, and anaemia, among children under five in Indonesia?’ This question was structured using the Population, Exposure, Comparator, Outcomes, and Study Design (PECOS) framework, as summarised in Table 1.

**Table 1.** Definition of Population, Exposure, Comparators, Outcomes, and Study Designs (PECOS) for the systematic review, 2025

PECOS		Definition
P	Population	Children under 5 years of age in Indonesia
E	Exposure	Household food security status
C	Comparison	Food-secure *v*. food-insecure households
O	Outcome	Prevalence of stunting, underweight, wasting, overweight, obesity and anaemia
S	Study design	Quantitative, qualitative or mixed-method research

### Eligibility criteria

Table 1 presents the definition of each element in the PECOS framework used to guide the selection and evaluation of studies in this systematic review. This framework was applied to ensure a clear and structured approach in identifying relevant evidence on the association between HFS and malnutrition among children under 5 years of age in Indonesia.

To ensure the relevance, rigour and contextual appropriateness of the studies included in this systematic review, a set of clearly defined inclusion and exclusion criteria was applied. A detailed summary of these parameters is presented in Table 2. Although policy relevance was not employed as a formal inclusion criterion during the selection process, studies that provided insights or implications for nutrition-related policy or household-level food security interventions were noted to enhance the interpretive and translational value of the review findings.

**Table 2.** Criteria for inclusion and exclusion of literature sources selected for review

Criteria	Inclusion	Exclusion
Publication type	Peer-reviewed journal articles and peer-reviewed conference proceedings published between 2015 and 2025	Non-peer-reviewed publications, editorials, opinion pieces, letters to editors and theses/dissertations
Language	Published in English or Bahasa Indonesia	Publications in other languages without available English/Indonesian translation
Population	Children aged 0–59 months (under 5 years of age) residing in Indonesia	Studies involving older children, adults or populations outside Indonesia
Study focus	Explicitly investigates the relationship between household food security (HFS) and at least one malnutrition outcome: stunting, underweight, wasting, overweight or anaemia	Studies not addressing food security or not reporting nutritional outcomes
Study design	Quantitative (cross-sectional, case–control, cohort), qualitative, or mixed-method studies	Reviews, purely descriptive reports, commentaries, or studies with unclear methodology
Geographic scope	Studies conducted in Indonesian settings (national or regional)	Multi-country studies without separate disaggregated data for Indonesia

### Search strategy

A comprehensive and systematic search strategy was formulated to retrieve peer-reviewed literature directly relevant to the research question. Searches were carried out in some major databases: Scopus, PubMed, ScienceDirect and EBSCO. In addition, grey literature was explored through ProQuest and Google Scholar (with the search limited to the top 200 most relevant results in the initial search and the top 100 in the updated search). The article search commenced on 10 March–13 April 2025. The Boolean search terms used included:

1. (‘Undernutrition’ OR ‘Overnutrition’ OR ‘Deficiency Micronutrient’) AND (‘Child’ OR ‘Children’ OR ‘Under Five Years’) AND (‘Indonesia’) AND (‘Food Security’)
2. (‘Stunting’ OR ‘Wasting’ OR ‘Underweight’ OR ‘Overweight’ OR ‘Obese’ OR ‘Anaemia’) AND (‘Child’ OR ‘Children’ OR ‘Under Five Years’) AND (‘Indonesia’) AND (‘Food Security’)
3. (‘Ketahanan Pangan Rumah Tangga’ OR ‘Ketahanan Pangan’) AND (‘Kekurangan Gizi’ OR ‘Kelebihan Gizi’ OR ‘Defisiensi Mikronutrien’) AND (‘Balita’) AND (‘Indonesia’)
4. (‘Ketahanan Pangan Rumah Tangga’ OR ‘Ketahanan Pangan’) AND (‘Stunting’ OR ‘Wasting’ OR ‘Underweight’ OR ‘Overweight’ OR ‘Obese’ OR ‘Anaemia’) AND (‘Balita’) AND (‘Indonesia’)

### Article selection

**Figure 1.**
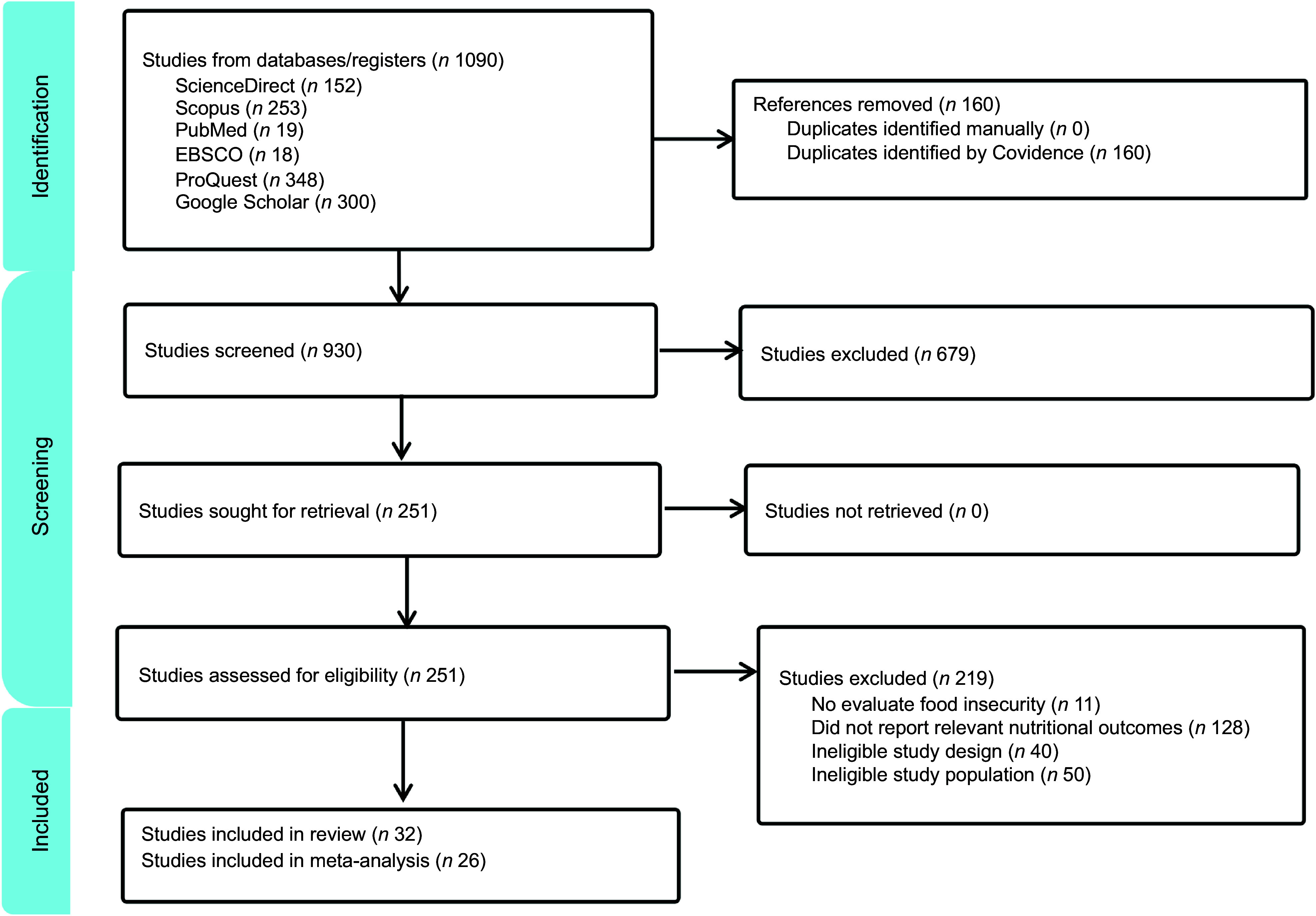
Flow chart for the study selection process.

All identified peer-reviewed and grey literature sources found using selected databases were uploaded to Covidence. Duplicate records were removed. Titles and abstracts were screened for relevance, followed by full-text screening against the inclusion and exclusion criteria. After all of the screening was done, thirty-two studies met the criteria for inclusion and exclusion and were selected for this review for a detailed analysis. The article screening was completed in April 2025. Figure 1 shows the flow chart for the study selection process.

### Assessment of methodological quality

The methodological quality of all included studies was independently assessed using the Joanna Briggs Institute (JBI) critical appraisal tools specific to cross-sectional and case–control study designs (see online supplementary material, Supplemental 1). For cross-sectional studies, the appraisal tool included eight domains covering the clarity of inclusion criteria, identification and management of confounding factors (marked not applicable for purely descriptive studies), validity and reliability of exposure and outcome measurements and appropriateness of statistical analyses. The latter was considered adequate if the study reported the statistical tests used, the software applied and the level of significance. For case–control studies, the JBI tool comprised ten items assessing criteria such as case and control definition, comparability of groups, exposure measurement, handling of confounding variables and the appropriateness of statistical analysis. Each item was rated as ‘Yes’, ‘No’, ‘Unclear’ or ‘Not Applicable’. Results from the quality assessment informed the interpretation of findings but were not used to exclude studies.

### Data extraction and synthesis

Data from eligible studies were extracted using a standardised data extraction form. The following variables were recorded:

- Author(s) and publication year
- Study design and methodology
- Population characteristics (age, number of samples and location)
- Indicators of HFS
- Types of malnutrition outcomes reported
- Main findings and conclusions

The data were tabulated for synthesis and analysed thematically to identify the association between food security and malnutrition. Studies were categorised by types of malnutrition addressed. Key policy interventions were also recorded to inform the implications section of the review.

A narrative synthesis approach was employed to summarise and interpret findings across the included studies. Results were divided into five groups thematically by malnutrition outcomes (stunting, underweight, wasting, overnutrition and anaemia). Tables and matrices were used to facilitate cross-study comparisons.

### Data analysis

Descriptive information of the included studies was compiled, including study design, sample size, population characteristics (age group), setting, type of food insecurity measurement tool used and nutritional outcomes assessed (stunting, wasting, underweight, overweight/obesity and anaemia). The primary outcomes were the associations between HFI and nutritional indicators, including stunting, wasting, underweight, overnutrition (overweight/obesity) and anaemia. For each included study, effect sizes were extracted as OR and their corresponding 95 % CI. Effect sizes were synthesised using generic inverse variance methods when available (e.g. for adjusted OR). If OR and 95 % CI were not directly reported in the original studies, raw data were extracted to construct 2 × 2 contingency tables, and unadjusted OR with corresponding 95 % CI were calculated manually.

Meta-analyses were performed when at least two studies reported comparable effect measures that could be pooled. Studies reporting insufficient data to calculate effect estimates were excluded from quantitative synthesis but were retained in the narrative synthesis. Meta-analyses were conducted separately for each outcome (stunting, wasting, underweight, overweight/obesity and anaemia), and where necessary, sensitivity analyses were conducted by excluding studies with extreme effect sizes or high risk of bias. Publication bias was assessed using a funnel plot for outcomes with ≥ 10 studies.

Random-effects models using the DerSimonian and Laird method were employed to calculate pooled OR and 95 % CI, considering anticipated heterogeneity across study settings, measurement tools and populations. Statistical heterogeneity was evaluated using the *I* ^2^ statistic, with *I* ^2^ values > 40·0 % considered indicative of moderate to substantial heterogeneity^(21)^. Funnel plots were inspected to assess potential publication bias where at least ten studies were available for the outcome. Meta-regression was not performed due to the limited number of studies per outcome. All statistical analyses were conducted using Review Manager (RevMan 5.4, The Cochrane Collaboration).

The certainty of the evidence for each meta-analysis was assessed using the Grading of Recommendations Assessment, Development, and Evaluation (GRADE) approach. This assessment considered the following domains: study limitations (risk of bias), inconsistency of results, indirectness of evidence, imprecision of estimates and potential publication bias.

Each outcome (stunting, wasting, underweight, overweight/obesity and anaemia) was graded individually based on these domains. The quality of evidence was categorised as high, moderate, low or very low. Observational studies started with a low level of evidence, which could be upgraded or downgraded depending on the criteria met.

## Result

A total of thirty-two studies met the inclusion criteria and were included in this systematic review, of which twenty-six studies were eligible for quantitative synthesis. The majority of included studies employed a cross-sectional design (*n* 25), while seven studies used a case–control design. Most studies were conducted at the subnational level, encompassing diverse geographical settings across Indonesia, including Java (*n* 18), Sumatra (*n* 8), Sulawesi (*n* 3) and Kalimantan (*n* 1), covering both urban and rural populations. In addition, two studies utilised nationally representative datasets, such as the Indonesia Family Life Survey (IFLS) and the Indonesian Nutritional Status Survey, providing broader population coverage.

HFI was assessed using a range of validated measurement tools, including the US Household Food Security Survey Module (US-HFSSM), the Household Food Insecurity Access Scale (HFIAS), the Household Dietary Diversity Score (HDDS), the Food Insecurity Experience Scale (FIES), the Food Consumption Score (FCS) and locally adapted food security indices. The nutritional outcomes assessed encompassed multiple forms of malnutrition, namely stunting, underweight, wasting, overweight/obesity and anaemia, allowing a comprehensive examination of the association between HFI and child nutritional status across different epidemiological and geographical contexts in Indonesia. Overall, HFI was associated with multiple forms of malnutrition among Indonesian children under 5 years of age, with pooled OR ranging from 0·41 to 5·26, and the strongest association observed for underweight.

### Food insecurity and stunting

We included seven case–control studies from 2018 to 2024 to examine the correlation between HFI and stunting in Indonesian children under 5 years of age^(22–28)^. A summary of the main features and conclusions of these case–control studies is provided in online supplementary material, Supplemental 2 (Table 2A). A statistically significant correlation between stunting in children under 5 years of age and HFI was consistently shown in seven case–control studies.

There was a consistent pattern of higher food insecurity in the stunted group, with prevalences ranging from 29·6 % to 71·4 % among stunted children and from 25·0 % to 28·6 % among non-stunted controls across the studies. According to the North Sumatra study by Frisnoiry *et al.* ^(23)^, after controlling for maternal height and low birth weight, the odds of having stunted children were 2·62 times higher for households experiencing food insecurity (95 % CI: 1·01, 7·21, *P* = 0·050). Similarly, Raharja *et al.* ^(27)^ in Yogyakarta used a custom composite HFI index and discovered an even stronger association (OR = 3·16; 95 % CI: 1·33, 7·52, *P* = 0·007). Even using a non-standardised tool, Rohmawati *et al.* ^(28)^ found the highest effect estimate, with food-insecure households having more than threefold higher odds of child stunting (OR = 3·51; 95 % CI: 1·44, 8·56, *P* = 0·005). A statistically significant association (*P* = 0·041) was reported by Fadzila and Tertiyus ^(22)^ using US-HFSSM in East Java, where 41·7 % of stunted children lived in food-insecure households, compared to 25·0 % among controls. Wardani *et al.* ^(25)^ employed HFIAS in Lampung and found a strong correlation (C = 0·42, *P* < 0·001). Despite differences in methodology, every study produced statistically significant results (*P* < 0·050), supporting the idea that HFI is a strong and independent risk factor for stunting in young children across Indonesia’s various socio-economic and geographic contexts.

Apart from case–control studies, twenty cross-sectional studies in all were incorporated to investigate the relationship between HFI and stunting among Indonesian children under 5 years of age (see online supplementary material, Supplemental 2 (Table 2B))^(29–48)^. Even after controlling for important covariates including mother education, birth weight, dietary diversity and household income, the majority (*n* 15) of the twenty studies reported a statistically significant association between HFI and stunting (*P* < 0·050). Using an adjusted OR (AOR) of 10·90 (95 % CI: 1·80, 67·30), Utami and Dwi^(30)^ in West Java found, for instance, a significantly higher risk of stunting among children from severely food-insecure households. Widyaningsih *et al*.^(33)^ and Asparian *et al.* ^(34)^ also reported high multivariate OR (8·33 and 4·72, respectively), reinforcing the consistency and strength of this relationship across settings.

Although the association was not strong in urban settings, Masitoh *et al.* ^(41)^ examined national-level survey data from over 82 000 children and found that moderate and severe food insecurity considerably raised the prevalence of stunting in rural areas (aPR = 1·09 and 1·15, respectively). Similarly, Sanggelorang *et al.* ^(45)^, using subnational data from over 8000 children in North Sulawesi, noted a particularly strong association between household food vulnerability and stunting (OR = 4·66; 95 % CI: 4·19, 5·18).

Nonetheless, a small number of studies did not find statistically significant associations, such as Priawantiputri *et al.* ^(29)^, Gunawan and Septriana^(46)^ and Nashira *et al.* ^(44)^. These null results could be explained by particular local settings, measurement constraints or low variability in food security level within the study population.

### Food insecurity and underweight

A total of six studies also explored the association between HFI and underweight status among children under 5 years of age, as presented in online supplementary material, Supplemental 2 (Table 2C)^(29,38,40,42,49,50)^. Four of the six studies noted statistically significant links between underweight status and HFI. For example, Riski *et al.* ^(50)^ in Surabaya found a strong correlation between food insecurity and underweight (*r* = 0·46, *P* < 0·001), while Rifayanto *et al.* ^(38)^ reported a similar effect size (*r* = 0·50, *P* = 0·001). Particularly among fishing communities, Masthalina *et al.* ^(40)^ found a particularly noteworthy outcome, whereby children from food-insecure households had over six times higher odds of being underweight (OR = 6·30, *P* = 0·039). Additionally, Sutriningsih and Lastri^(49)^ investigated post-disaster households in Malang Regency and found a strong and statistically significant association between HFS and nutritional status (*r* = 1·00, *P* = 0·049), highlighting the vulnerability of children to undernutrition in disaster-prone settings.

Conversely, Priawantiputri *et al.* ^(29)^ and Adhyanti *et al.* ^(42)^ found no statistically significant association between HFI and underweight among children under 5 years of age. In Priawantiputri *et al.* ^(29)^, the prevalence of underweight was 15·9 %, with no meaningful difference across food security categories (*P* = 0·714). Similarly, Adhyanti *et al.* ^(42)^ reported an underweight prevalence of 18·7 % among disaster-affected households, yet the association with food security status was not significant (*P* = 0·202).

### Food insecurity and wasting

The association between HFI and wasting in children under 5 years of age is presented in online supplementary material, Supplemental 2 (Table 2D)^(29,38,40,42,51,52)^. From six cross-sectional studies, only Firmansyah *et al.* ^(51)^, using national data from the IFLS, reported that moderate and severe food insecurity significantly increased the odds of both wasting and severe wasting in children. With *P* = 0·001, the chances of wasting were specifically higher in mildly food-insecure households (OR = 2·02; 95 % CI: 1·55, 2·62) and severely food-insecure households (OR = 1·87; 95 % CI: 1·40, 2·62).

Food insecurity and wasting were not statistically significantly linked in the remaining six studies, Priawantiputri *et al.* ^(29)^, Sihotang and Rumida^(52)^, Rifayanto *et al*.^(38)^, Masthalina *et al.* ^(40)^ and Adhyanti *et al.* ^(42)^. For example, Rifayanto *et al.* ^(38)^ found no correlation (*r* = 0·05, *P* = 0·770), while Adhyanti *et al.* ^(42)^ also reported non-significant results in a post-disaster context (*P* = 0·253).

Overall, the findings suggest a less consistent relationship between HFI and wasting when compared to other undernutrition (stunting or underweight). This may be attributed to the acute nature of wasting, which may be influenced more strongly by recent illness or infection rather than chronic food access issues alone.

### Food insecurity and overnutrition

A smaller number of studies investigated the association between HFI and overnutrition among children under 5 years of age. Three cross-sectional studies assessed this relationship using nationally representative datasets and local surveys across various regions in Indonesia (see online supplementary material, Supplemental 2 (Table 2E)^(48,51,52)^.

Using data from 4391 children in the IFLS, Firmansyah *et al.* ^(51)^ showed that moderate and severe food insecurity were significantly associated with increased odds of both overweight and obesity. Specifically, moderately food-insecure children had 1·589 times higher odds of being overweight (95 % CI: 1·15, 1·83) and 2·22 times higher odds of being obese (95 % CI: 1·58, 3·11), compared to food-secure peers. Similar elevated risks were observed for those classified as severely food-insecure (*P* < 0·050 for all).

In a localised study, Hidayati^(48)^ in East Java also found a statistically significant association between HFS status and weight-for-height classification among children under 5 years of age (*P* = 0·042), with a combined prevalence of 13·0 % for overweight and 4·0 % for obesity. On the other hand, Sihotang and Rumida^(52)^ did not find a statistically significant association between food insecurity and overweight among toddlers in Deli Serdang Regency (*P* = 0·488), despite a reported overweight prevalence of 12·8 %.

Overall, while fewer studies addressed overnutrition compared to undernutrition outcomes, the majority of available data indicate a significant association between HFI and increased risk of overweight and obesity among Indonesian children under 5 years of age, suggesting the potential coexistence of food insecurity and excessive energy intake in some vulnerable populations.

### Food insecurity and anaemia

Two cross-sectional studies examining the association between HFI and anaemia among children under 2 years of age in Indonesia are presented in online supplementary material, Supplemental 2 (Table 2F). Both studies reported statistically significant associations, indicating that children living in food-insecure households were more likely to be anaemi^(29,53)^.

In Rohmah *et al.* ^(53)^, conducted in Karawang Regency, West Java, food insecurity was associated with significantly higher odds of anaemia (OR = 4·85; 95 % CI: 1·79, 13·15; *P* = 0·003). This association remained robust in the multivariate model (AOR = 10·05; *P* = 0·003), even after controlling for maternal age, education, employment status, knowledge about anaemia and parenting practices. Similarly, Priawantiputri *et al.* ^(29)^ found a significant association between food insecurity with hunger and anaemia (*P* = 0·037), with an overall anaemia prevalence of 63·6 % among children under 2 years of age in Kolaka Timur Regency.

These findings highlight the potential role of food insecurity not only in contributing to anthropometric deficits but also in increasing the risk of micronutrient deficiencies, particularly Fe deficiency anaemia. Such evidence underscores the importance of incorporating nutrition-sensitive components into HFS interventions targeting vulnerable infants and young children.

### Meta-analysis

**Figure 2.**
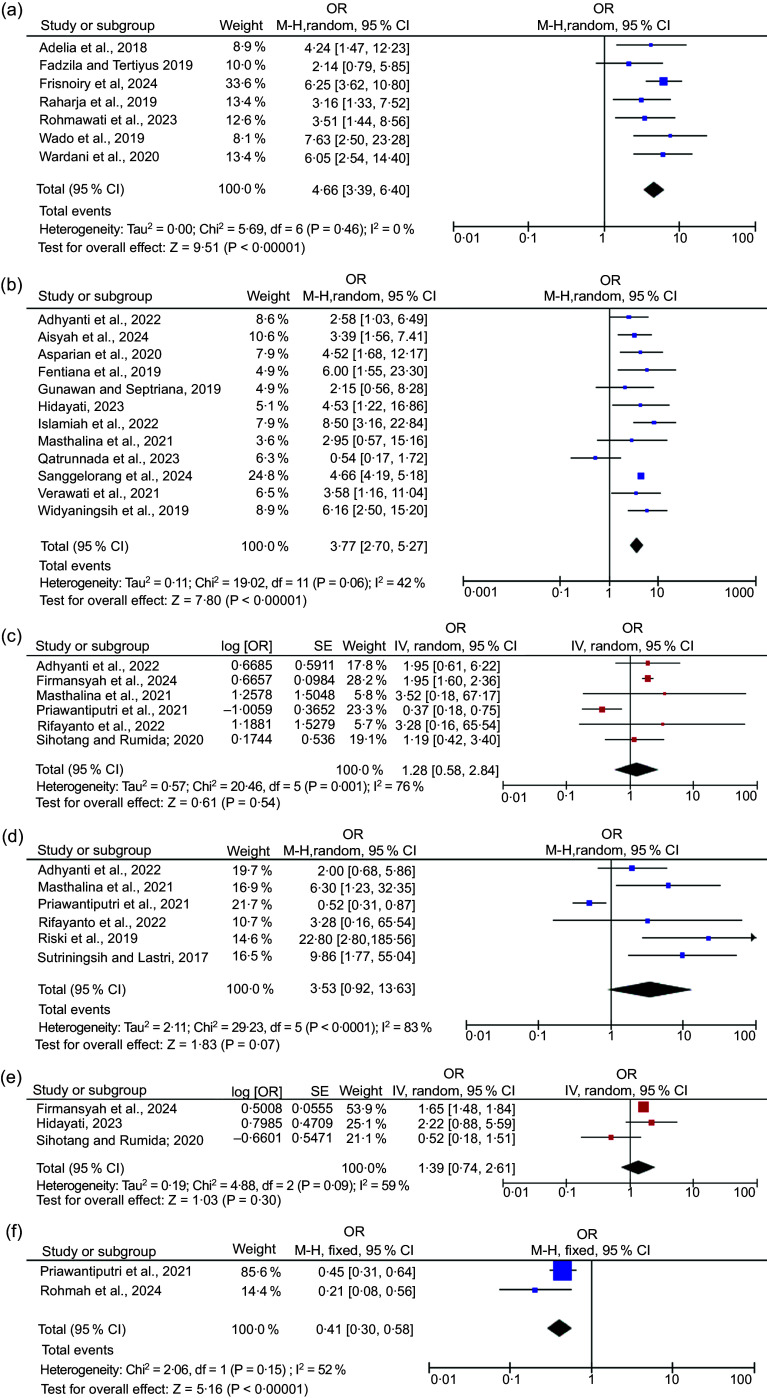
Forest plot of the association between HFI and child malnutrition. (a) Association between HFI and stunting in children under 5 years of age from case–control studies (*n* 7). (b) Association between HFI and stunting in children under 5 years of age from cross-sectional studies (*n* 12). (c) Association between HFI and wasting in children under 5 years of age (*n* 6). (d) Association between HFI and underweight among children under 5 years of age (*n* 6). (e) Association between HFI and overnutrition in children under 5 years of age (*n* 3). (f) Association between HFI and anaemia in children under 5 years of age (*n* 2). HFI, household food insecurity.

To quantify the strength of associations reported in the systematic review, we performed meta-analyses for each outcome domain using eligible cross-sectional and case–control studies. The results are presented in Figure 2, corresponding to the main nutritional outcomes: stunting, wasting, underweight, overnutrition and anaemia.

The case–control studies were analysed separately, and the forest plot in Figure 2(a) shows a significant association between HFI and the risk of stunting among children under 5 years of age. The pooled OR was 4·66 (95 % CI: 3·39, 6·40, *P* < 0·001), with no observed heterogeneity across studies (*I* ^2^ = 0·0 %).

**Figure 3.**
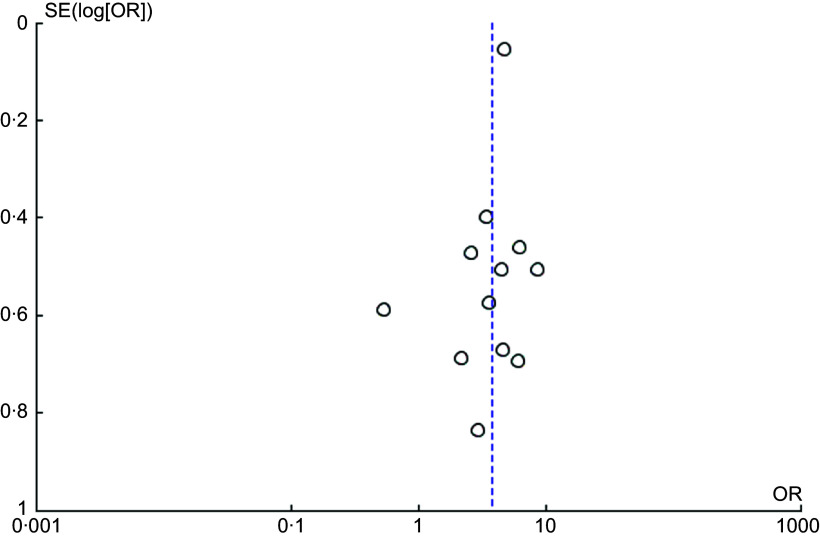
Funnel plot assessing publication bias for the association between household food insecurity and stunting (cross-sectional studies).

Figure 2(b) shows the pooled effect from twelve cross-sectional studies examining the relationship between HFI and stunting. Children living in food-insecure households were 3·77 times more likely to be stunted (OR = 3·77; 95 % CI = 2·70, 5·27; *P* < 0·001). Moderate heterogeneity was observed (*I* ^2^ = 42·0 %). Visual inspection of the funnel plot for cross-sectional studies on stunting suggested no substantial asymmetry, indicating low risk of publication bias (Figure 3). To test the robustness of this finding, a sensitivity analysis was conducted by excluding an outlier study Qatrunnada *et al.* ^(36)^ and demonstrated a strong and consistent association between HFI and stunting in children under 5 years of age (OR = 4·61; 95 % CI: 4·17, 5·11; *I* ^2^ = 0·0 %; *P* < 0·001). This suggests a fourfold increased risk of stunting in children living in food-insecure households.

For wasting, six cross-sectional studies were included in the meta-analysis (Figure 2(c)), revealing a positive association with HFI yielded a pooled OR of 1·28 (95 % CI: 0·58, 2·84; *P* = 0·540; *I* ^2^ = 76·0 %), indicating a non-significant association and substantial heterogeneity. Only one study from Firmansyah *et al.* ^(51)^ reported a statistically significant positive association. Notably, one study from Priawantiputri *et al.* ^(29)^ reported an inverse association, contributing to the overall heterogeneity. Excluding Priawantiputri *et al.* ^(29)^ due to sensitivity analysis, reported a significant and consistent pooled estimate (OR = 1·92; 95 % CI: 1·60, 2·32; *P* < 0·001), with no observed heterogeneity (*I* ^2^ = 0·0 %).

Figure 2(d) presents the meta-analysis of six studies assessing the relationship between HFI and underweight among children under 5 years of age, producing a pooled OR of 3·53 (95 % CI: 0·92, 13·63; *P* = 0·070), indicating a non-significant but positive trend. However, heterogeneity was substantial (*I* ^2^ = 83·0 %), reflecting differences in effect directions and study designs. One study^(29)^ reported an inverse association (OR < 1·00), contributing to overall heterogeneity. A sensitivity analysis was conducted by excluding the study of Priawantiputri *et al.* ^(29)^. The revised analysis showed a significant and stronger association between HFI and underweight (OR = 5·26; 95 % CI: 2·12, 13·04; *P* < 0·001), with a notable decrease in heterogeneity (*I* ^2^ = 28·0 %).

For overnutrition outcomes, including overweight and obesity, the meta-analysis included three studies (Figure 2(e)). Three studies assessing the relationship between HFI and overnutrition yielded a pooled OR of 1·39 (95 % CI: 0·74, 2·61; *P* = 0·300), indicating a non-significant association. Moderate heterogeneity was detected (*I* ^2^ = 59·0 %). While two studies showed a positive association, one study reported an inverse effect, contributing to inconsistency in the pooled analysis. The sensitivity analysis excluding Sihotang and Rumida^(52)^. After removing that study, the pooled analysis revealed a significant association between food insecurity and overnutrition (OR = 1·66; 95 % CI: 1·49, 1·85; *P* < 0·001), with no observed heterogeneity (*I* ^2^ = 0·0 %).

Finally, the pooled effect from two studies examining anaemia in young children (< 2 years) is shown in Figure 2(f). A significant inverse association between HFS and anaemia among children under 5 years of age is revealed (OR = 0·41; 95 % CI: 0·30, 0·58; *P* < 0·001). This suggests that children from food-secure households were approximately 60 % less likely to experience anaemia compared to those from food-insecure households. Moderate heterogeneity was observed (*I* ^2^ = 52·0 %).

Taken together, the meta-analysis confirms a significant and consistent association between HFI and stunting, while the associations with underweight and overnutrition were more variable but remained significant after sensitivity analysis. The findings underscore the multidimensional nature of malnutrition linked to food insecurity, reinforcing the need for multisectoral interventions.

### Certainty of evidence

The certainty of evidence for each meta-analysis was assessed using the GRADE approach, considering risk of bias, inconsistency, indirectness, imprecision and publication bias. The overall certainty was rated as moderate to high for the associations between HFI and stunting (both cross-sectional and case–control) and anaemia, supported by large sample sizes, narrow CI and consistent findings across studies. The evidence for wasting and underweight was rated as moderate, primarily due to high heterogeneity (*I* ^2^ > 75·0 %) and imprecision in some effect estimates. For overnutrition (overweight and obesity), the certainty was also rated as moderate, due to the limited number of studies and minor imprecision, despite consistent effect direction. No serious concerns were identified regarding publication bias in any of the analyses. A summary of the GRADE assessments is presented in online supplementary material, Supplemental 3.

## Discussion

This systematic review and meta-analysis confirm that HFI is a significant and independent risk factor for stunting among children under 5 years of age in Indonesia. All seven case–control studies and the majority of cross-sectional studies demonstrated consistent and statistically significant associations between HFI and stunting, even after adjusting for important covariates such as maternal education, birth weight and dietary diversity^(30,33)^. The pooled analysis of twelve cross-sectional studies revealed that children from food-insecure households were 3·77 times more likely to be stunted (OR = 3·77; 95 % CI: 2·70, 5·27), while case–control data yielded a pooled OR of 4·66 (95 % CI: 3·39, 6·40), both with negligible heterogeneity after sensitivity adjustments. These findings are in line with global literature that identifies inadequate access to sufficient, safe and nutritious food as a fundamental driver of linear growth failure during the first 1000 d of life^(54,55)^.

The magnitude of the association was particularly pronounced in rural and marginalised areas such as Gunungkidul and East Nusa Tenggara, where poverty, limited infrastructure and poor health services exacerbate food insecurity’s impact on child growth^(56)^. However, several urban studies, including those from Surabaya and Jakarta, reported similarly high stunting risks among children from food-insecure households, suggesting that urban slums face their own unique vulnerabilities – such as high food prices, lack of dietary diversity and informal housing conditions^(32)^. National survey data also show that the association between HFI and stunting is stronger in rural areas^(41)^, though not absent in urban contexts. This pattern underscores the role of structural determinants – such as sanitation, maternal education and access to health services – that interact with food insecurity to influence child nutritional status^(19,45)^.

Although standardised instruments like HFIAS and FIES were widely used, they may not fully capture culturally embedded dynamics such as intra-household food allocation, seasonal food insecurity or dietary taboos, which can disproportionately affect young children^(57,58)^. Therefore, future research should complement quantitative assessments with qualitative, context-sensitive approaches to improve the precision and relevance of HFI measurement. From a policy perspective, these findings affirm the need for integrated, multisectoral strategies that combine nutrition-specific interventions (e.g. supplementation, growth monitoring) with nutrition-sensitive approaches, including maternal education, social protection, local food system strengthening and WASH infrastructure improvements^(59)^. These interventions must be tailored to Indonesia’s diverse sociogeographic contexts to effectively reduce the long-term burden of stunting and achieve national nutrition targets.

While the association between HFI and stunting was consistent, several studies have also investigated how food insecurity may contribute to underweight among children under 5 years of age. Of the six included studies, four reported statistically significant associations, highlighting a consistent trend where children from food-insecure households were more likely to be underweight^(38,40,50)^. In fishing and disaster-prone communities, such as North Sumatra and Malang Regency, the risk appears to be magnified due to cyclical income instability, poor diet diversity and disrupted food systems. These findings are supported by global literature indicating that HFI is associated with low dietary adequacy, delayed growth and increased susceptibility to infections that compromise nutritional status^(55)^.

Two studies, however, did not find a statistically significant association^(29,42)^, potentially due to contextual or methodological differences such as homogenous food security levels, sample size limitations or post-disaster recovery stages. The high heterogeneity (*I* ^2^ = 83·0 %) observed in the meta-analysis further reflects these variabilities in population risk profiles and study design. Yet, once the most divergent study^(29)^ was excluded in sensitivity analysis, the pooled estimate became both statistically significant (OR = 5·26; 95 % CI: 2·12, 13·04; *P* < 0·001) and more consistent across studies (*I* ^2^ = 28·0 %), reaffirming the role of HFI as a meaningful risk factor for underweight.

These results underscore the importance of context-sensitive strategies for undernutrition reduction. Children in food-insecure households may not necessarily experience calorie deficiency, but rather inadequate intake of nutrient-dense foods – leading to chronic underweight without overt wasting. Therefore, addressing underweight in Indonesia demands interventions that improve both food access and dietary quality, particularly among vulnerable groups in coastal, low-income or post-disaster areas. This includes integrating HFS assessments into growth monitoring, diversifying food assistance programmes and strengthening nutrition education tailored to local cultural norms^(32,54)^.

In addition to underweight, wasting – an indicator of acute undernutrition – was assessed to examine whether HFI is linked with rapid weight loss or failure to gain weight in the short term. Among seven cross-sectional studies reviewed, only one – Firmansyah *et al.* ^(51)^, using nationally representative data from the IFLS – identified a statistically significant positive association. The odds of wasting were markedly higher in both moderately and severely food-insecure households, with adjusted OR above 1·8. In contrast, the other six studies, including those by Rifayanto *et al*.^(38)^, Priawantiputri *et al.* ^(29)^ and Adhyanti *et al.* ^(42)^, reported no significant association between food insecurity and wasting. These inconsistencies suggest that the relationship may be context-dependent and moderated by other factors such as recent illness episodes, sanitation or maternal caregiving practices.

This variability was reflected in the initial meta-analysis, which yielded a non-significant pooled OR of 1·28 (95 % CI: 0·58, 2·84; *P* = 0·540) and high heterogeneity (*I* ^2^ = 76·0 %). One study^(29)^ even reported an inverse relationship, which likely contributed to the inconsistency. After excluding this outlier in a sensitivity analysis, the association became statistically significant (OR = 1·92; 95 % CI: 1·60, 2·32; *P* < 0·001), and heterogeneity dropped to zero (*I* ^2^ = 0·0 %). This result strengthens the evidence that, while not universally observed, HFI may indeed elevate the risk of wasting, especially in large-scale or well-controlled studies with population diversity and rigorous measurement.

These findings align with broader global literature suggesting that wasting, as an acute form of undernutrition, is often more sensitive to short-term shocks – such as disease outbreaks, food scarcity due to seasonal factors or sudden income loss – than to chronic food insecurity^(55)^. Therefore, programmatic responses to wasting should combine both nutrition-specific interventions (e.g. treatment of acute malnutrition, infection control) and food security components that can stabilise household access to safe, diverse and nutrient-rich food. Integrated approaches are particularly critical in disaster-prone, urban poor or climate-vulnerable regions where both food access and health risks fluctuate rapidly.

Interestingly, food insecurity was not solely associated with nutritional deficits. Several studies have explored its link with childhood overweight and obesity, suggesting the emergence of a double burden of malnutrition. Among five cross-sectional studies, three found a statistically significant association between food insecurity and increased risk of overweight and obesity^(48,51)^. Firmansyah *et al.* ^(51)^, using a large national dataset from the IFLS, showed that children from moderately and severely food-insecure households had significantly higher odds of being both overweight and obese. This finding supports global trends indicating that economic hardship often drives families towards high-calorie, nutrient-poor food choices, contributing to excessive energy intake^(19)^.

The pooled analysis of three studies yielded an initial non-significant association (OR = 1·39; 95 % CI: 0·74, 2·61; *P* = 0·300), with moderate heterogeneity (*I* ^2^ = 59·0 %), likely due to one study^(52)^ reporting an inverse association. However, the sensitivity analysis that excluded this outlier demonstrated a statistically significant relationship (OR = 1·66; 95 % CI: 1·49, 1·85; *P* < 0·001) and eliminated heterogeneity (*I* ^2^ = 0·0 %). This suggests that, when methodological variability is accounted for, HFI is consistently linked to increased risk of overnutrition in children – reinforcing the ‘double burden of malnutrition’ framework, where both undernutrition and overnutrition coexist within the same population or household.

These findings imply that food insecurity is not only a marker of inadequate food quantity but also of poor diet quality. Households with limited financial resources may prioritise satiety over nutritional value, leading to increased consumption of energy-dense foods that are high in sugars and fats but low in essential nutrients. In urban poor and transitional rural settings, this may manifest as rising rates of childhood overweight even in households categorised as food-insecure. Addressing overnutrition in such contexts requires multifaceted interventions that combine food security strategies with nutrition education, regulation of unhealthy food marketing, and targeted subsidies for nutrient-dense foods^(32,54)^.

In addition to anthropometric outcomes, food insecurity may also impair micronutrient intake, leading to deficiencies such as Fe deficiency anaemia, especially in young children. This review reveals a significant association between HFI and the risk of anaemia among children under 2 years of age in Indonesia. Both included cross-sectional studies – Rohmah *et al.* ^(53)^ and Priawantiputri *et al.* ^(29)^ – demonstrated that children living in food-insecure households were significantly more likely to be anaemic. In particular, Rohmah *et al.* found that food-insecure households had nearly five times higher odds of child anaemia, with this association remaining significant even after adjusting for maternal characteristics and caregiving practices. These findings align with global evidence that food insecurity undermines access to Fe-rich foods and compromises dietary quality during critical growth periods^(55)^.

The meta-analysis supports these findings with a pooled OR of 0·41 (95 % CI: 0·30, 0·58; *P* < 0·001), indicating that children in food-secure households are approximately 60 % less likely to be anaemic than those in food-insecure households. Despite including only two studies, the results showed statistical significance and moderate heterogeneity (*I* ^2^ = 52·0 %), suggesting relatively consistent outcomes across settings. Given the high anaemia prevalence observed in both studies (over 60 % in Kolaka Timur), the findings underscore the urgent need to address micronutrient deficiencies – particularly Fe deficiency anaemia – as a key outcome of HFI.

The evidence highlights the necessity of incorporating nutrition-sensitive strategies within HFS programmes, especially for infants and young children during the first 1000 d of life. Interventions must move beyond calorie sufficiency to promote regular access to Fe-rich foods – such as fortified cereals, meat, legumes and leafy vegetables – and improve caregiver knowledge of anaemia prevention. Moreover, food assistance schemes like the Non-Cash Food Assistance Program (*Bantuan Pangan Non Tunai*/BPNT) should integrate micronutrient-rich components to reduce the risk of hidden hunger in food-insecure households. Addressing anaemia through household-level food security could significantly contribute to reducing early childhood morbidity and long-term developmental risks in Indonesia^(54)^.

Taken together, the meta-analysis confirms a robust and statistically significant association between HFI and stunting among children under 5 years of age in Indonesia, while associations with underweight and overnutrition were less consistent but became significant after sensitivity analyses. These findings reinforce the multidimensional nature of malnutrition, where food insecurity contributes not only to chronic growth faltering but also to inadequate weight gain and even excessive weight, depending on contextual factors such as diet quality, socio-economic conditions and access to health services. The coexistence of undernutrition and overnutrition within food-insecure populations highlights the ‘double burden of malnutrition’ and calls for integrated, multisectoral strategies that simultaneously address caloric adequacy, micronutrient intake and food quality. Effective interventions must go beyond food quantity and include maternal education, WASH (Water Sanitation and Hygiene) infrastructure, social protection and regulation of unhealthy food environments to disrupt the cyclical impact of poverty and food insecurity on child nutritional outcomes^(32,54)^.

In light of these findings, national and local governments in Indonesia must adopt a dual-track strategy, combining emergency responses to acute undernutrition and long-term investment in sustainable food systems, to protect children’s growth and development in both rural and urban food-insecure settings.

### Strengths and limitations

The study has several strengths. First, it is one of the few reviews to integrate both narrative synthesis and quantitative meta-analysis to examine the association between HFI and multiple nutritional outcomes in the Indonesian context. Second, it covers a recent and policy-relevant period (2015–2025), capturing current demographic, economic and nutritional transitions. The combined analytical approach enhances the robustness and generalisability of the findings. Compared with previous global meta-analyses, this review provides greater contextual specificity, thereby increasing its relevance for Indonesian policymakers.

However, several limitations should be acknowledged. The quality and consistency of the included studies varied, particularly with respect to the measurement of HFI and nutritional outcomes. A range of validated food insecurity instruments (e.g. HFIAS, US-HFSSM, HDDS, FIES, FCS and locally adapted tools) were used across studies, which may have contributed to heterogeneity in effect estimates. To address this variability, random-effects meta-analysis was employed, as this approach explicitly accounts for between-study heterogeneity in exposure measurement, study populations and analytical methods. In addition, sensitivity analyses were conducted to examine the influence of individual studies on pooled estimates. These analyses showed that heterogeneity was reduced after excluding studies that contributed most to between-study variability, while the pooled estimates remained statistically significant. This indicates that, although certain studies influenced the degree of heterogeneity, the overall conclusions were robust despite differences in measurement instruments.

Furthermore, many included studies relied on cross-sectional designs and lacked comprehensive adjustment for potential confounders, limiting causal inference. Variations in population characteristics and study settings also constrained comparability across studies. Publication bias cannot be entirely excluded due to limited access to grey literature and unpublished data. Finally, relatively few studies examined overnutrition and anaemia, highlighting the need for more focused research on these outcomes in the Indonesian context.

### Conclusions

This systematic review and meta-analysis confirm a significant association between HFI and multiple forms of child malnutrition in Indonesia, particularly stunting, underweight, wasting, overnutrition and anaemia. These findings underscore the multifaceted nature of malnutrition and the importance of addressing food insecurity as a critical determinant of child health. Given the persistent prevalence of malnutrition across Indonesian settings, targeted, context-specific interventions that enhance food access, dietary quality and household resilience are urgently needed. Strengthening food security policies and integrating nutrition-sensitive strategies into national programmes may contribute to reducing both undernutrition and overnutrition among children under 5 years of age.

## Supporting information

10.1017/S1368980026102365.sm001 Sutrisno et al. supplementary material 1 Sutrisno et al. supplementary material

10.1017/S1368980026102365.sm002 Sutrisno et al. supplementary material 2 Sutrisno et al. supplementary material

10.1017/S1368980026102365.sm003 Sutrisno et al. supplementary material 3 Sutrisno et al. supplementary material

## Supplementary material

For supplementary material accompanying this paper visit https://doi.org/10.1017/S1368980026102365
