# Supplementary material for: The role of household food insecurity in malnutrition among Indonesian children under 5 years of age: a systematic review and meta-analysis (2015–2025)
Source: Public Health Nutr. 2026 Mar 26;29(1):e86. doi: 10.1017/S1368980026102365 (PMC13112310; doi:10.1017/S1368980026102365)
Supplement: Sutrisno et al. supplementary material 1 — Sutrisno et al. supplementary material [file S1368980026102365sup001.docx]

**Supplementary Table 2A. Association Between HFS and Stunting (Case-Control Studies)**

| Author | Location | Participants | HFI Tool | Prevalence of HFI | Main result | JBI Quality |
| --- | --- | --- | --- | --- | --- | --- |
| Fadzila and Tertiyus 2019^22^ | Ngajuk Regency, East Java | 72 children aged 6–23 months  (36 stunted, 36 non-stunted) | United Stated-Household Food Security Survey Module (US-HFSSM) | - Total food insecurity: 33.3% of households - Among stunted: 41.7% were food insecure - Among non-stunted: 25.0% were food insecure | - Significant association between household food insecurity and stunting (*P* = 0.041) | Include (8/10) |
| Frisnoiry et al., 2024^23^ | Asahan Regency, North Sumatra | 252 children aged 6–23 months  (126 stunted, 126 controls) | Instrument developed by Radimer et al., includes household-level food access and affordability | - Stunted Children:   Food insecure: 71.4%   - Control group: - Food insecure: 28.6% | - Bivariate analysis: Food insecurity associated with stunting (*P* = 0.040; OR = 2.70; 95% CI: 1.04–7.00) - Multivariate analysis (adjusted for maternal height and LBW): food insecurity remained a significant risk factor (OR = 2.62; 95% CI: 1.01–7.21; *P* = 0.050) | Include (9/10) |
| Wado et al., 2019^24^ | North Semarang, Central Java | 160 children aged 1-5 years  (80 stunted and 80 normal) | Measured using combination of calorie intake and household food expenditure share to classify food security into 4 categories: food secure, less secure, vulnerable, and food insecure | - Food Insecure: 54.3% - Food Vulnerable: 6.2% - Less Secure: 22.8% - Food Secure: 16.7% | - Significant association between household food security and stunting using Spearman correlation test (*P* < 0.001). | Include (7/10) |
| Wardani et al., 2020^25^ | Bandar Lampung City, Lampung | 100 children aged 12–59 months  (50 stunted, 50 non-stunted) | Household Food Insecurity Access Scale (HFIAS) | - Food secure: 47.0% - Mild insecurity: 23.0% - Moderate: 18.0% - Severe: 12.0% | - Significant association between household food insecurity and stunting (*P* < 0.001; C = 0.42) | Include (8/10) |
| Adelina et al., 2018^26^ | Semarang Regency, Central Java | 70 children under five aged 24-59 months  (35 stunted, 35 normal) | Household Food Security assessed using a Food Frequency Questionnaire (FFQ) and Desirable Dietary Pattern (DDP) Score | - Stunted Children:   Food secure: 70.4%  Food insecure: 29.6%   - Normal Children: Food secure: 74.3% Food insecure: 25.7% | - Significant association between household food insecurity and stunting *P* = 0.049, OR = 3.06) | Include (7/10) |
| Raharja et al., 2019^27^ | Gunungkidul Regency, Special Region of Yogyakarta | 141 children aged 24–59 months (47 stunted, 94 non-stunted) | Custom household food security questionnaire based on: - % of food expenditure - access to food - family size  Categorized as: - Food secure: ≤60% food expenditure, easy access, ≤4 family members - Food insecure: >60% food expenditure, difficult access, >4 family members | - 68.1% food insecure households | - Significant association between household food insecurity and stunting: OR = 3.16 (95% CI: 1.33–7.52), *P* = 0.007 | Include (9/10) |
| Rohmawati et al., 2023^28^ | Pamekasan Regency, East Java | 88 children aged 24–59 months  (44 stunted, 44 non-stunted) | Structured questionnaire (validated and tested) completed by mothers to assess household food security (specific tool not named in the paper) | - 56.8% of stunted children lived in food-insecure households - 27.3% of non-stunted children lived in food-insecure households | - Food-insecure households had a significantly higher odds of stunting (OR = 3.51, 95% CI = 1.44–8.56, *P* = 0.005). | Include (8/10) |

**Supplementary Table 2B. Association Between HFS and Stunting (Cross sectional Studies)**

| **Author** | **Location** | **Participants** | **HFI Tool** | **Prevalence of HFI** | **Prevalence of Outcome** | **Main result** | **JBI Quality** |
| --- | --- | --- | --- | --- | --- | --- | --- |
| Priawantiputri et al., 2021^29^ | Kolaka Timur Regecy, Southeast Sulawesi | 429 children aged <2 years with their mothers | US-HFSSM – short version | - High or marginal food secure: 53.80% - Food insecure without hunger: 34.0% - Food insecure with hunger: 11.4% | Stunting: 20.5%, | - No significant association between household food security and Stunting (*P* = 0.940) | Include (7/8) |
| Utami and Dwi., 2015^30^ | Bogor City, West Java | 216 children (6-23 months) | HFIAS | - Food Secure (63.0%) - Mild food insecurity (17.0%) - Moderate (11.0%) - Severe (9.0%) | Food Secure:   - Stunted: 13.4% - Normal: 86.6%   Mild Food Insecurity:   - Stunted: 33.3% - Normal: 66.7%   Moderate Food Insecurity:   - Stunted: 33.3% - Normal: 66.7%   Severe Food Insecurity:   - Stunted: 40.0% - Normal: 60.0% | - Significant association between household food security and child nutritional status (*P* < 0.010). AOR = 10.90 (95% CI: 1.80–67.30), after adjusting for covariates like child's age, birth weight, breastfeeding practices, use of feeding bottles, dietary diversity, mother's age, mother's education, mother's nutrition knowledge, food hygiene practices, father's education, and father's occupation | Include (8/8) |
| Gunawan and Septriana, 2019^46^ | Gunungkidul Regency, Special Region of Yogyakarta | 73 children aged 6–59 months | HFIAS | - Food secure: 13.7% - Mild food insecurity: 43.8% - Moderate food insecurity: 24.7% - Severe food insecurity: 17.8% | Stunted: 65.8% Normal: 34.2% | - No significant association between household food security and stunting (*P* = 0.258) | Include (6/8) |
| Islamiah et al., 2022^47^ | Pasuruan City, East Java | 87 households with toddlers (children aged 6–59 months) from fisherman families | HFIAS | - Severe Food Insecurity: 16.1% - Moderate Food Insecurity: 35.6% - Food Secure: 48.3% | Child stunting: 43.7% | - Significant associations were found between household food security status and stunting (*P* = 0.000) - Stunting risk was higher among households categorized as moderately or severely food insecure compared to food-secure households | Include (7/8) |
| Hidayati, 2023^48^ | Pasuruan Regency, East Java | 100 children under five years (6–59 months) | US-HFSSM | - High food security: 42.0% - Marginal food security: 47.0% - Low food security: 9.0% - Very low food security: 2.0% | Food Secure:   - Severely stunted: 2 children (4.8%) - Stunted: 1 child (2.4%) - Normal: 39 children (92.8%) - Tall: 0 children (0.0%)   Food insecure without hunger (n = 47):   - Severely stunted: 4 children (8.5%) - Stunted: 7 children (14.9%) - Normal: 34 children (72.3%) - Tall: 2 children (4.3%)   Moderate hunger (n = 9):   - Severely stunted: 0 children (0.0%) - Stunted: 4 children (44.4%) - Normal: 5 children (55. 6%) - Tall: 0 children (0.0%)   Severe hunger (n = 2):   - Severely stunted: 0 children (0.0%) - Stunted: 0 children (0.0%) - Normal: 1 child (50.0%) - Tall: 1 child (50.0%) | - There is a statistically significant association between household food security status and child height-for-age nutritional status (p = 0.013) | Include (7/8) |
| Safitri and Nindya, 2017^31^ | Surabaya City, East Java | 68 children aged 13–48 months | US-HFSSM | - Food Insecurity: 61.8% | Stunting: 30.9% | - There is a significant association between household food insecurity and stunting (*P* = 0.001) | Include (7/8) |
| Mahmudiono et al., 2018^32^ | Urban areas of Surabaya, East Java | 685 mother–child pairs (children aged 2–5 years) | HFIAS | - Food secure: 42.0% - Mildly insecure: 22.9% - Moderately insecure: 15.3% - Severely insecure: 19.7% | Child stunting: 36.5% | - Severe Food Insecurity significantly associated with child stunting aOR = 2.01 (1.14–3.53); *P* = < 0.05 | Include (8/8) |
| Widyaningsih et al., 2019^33^ | Klaten Regency, Central Java | 100 toddlers (24-59 months) | Measured by Household Adequacy Level of Energy using 24-hour recall. Households consuming <70% of energy requirements were considered food insecure. | - 35.0% households were food insecure | 41.0% of toddlers were stunted | - Bivariate analysis: Household food insecurity was significantly associated with stunting (OR = 6.16, 95% CI: 2.50–15.20, *P* = 0.000) - Multivariate analysis: Household food insecurity was significantly associated with stunting in toddlers (OR = 8.33, 95% CI: 2.86–24.25, *P* = 0.000), after adjusting for birth weight, birth length, maternal education, and food diversity | Include (8/8) |
| Asparian et al., 2020^34^ | Kerinci Regency, Jambi | 98 toddlers aged 24–59 months from farmer households | Household Dietary Diversity Score (HDDS) | - Low food security: 26.5% - High food security: 63.5% | Stunting prevalence was 46.9% among toddlers | - Food insecurity was significantly associated with stunting (PR = 2.32 (1.20 - 4.48), *P* = 0.004). - Multivariate analysis confirmed food security as the most dominant factor (OR = 4.72; 95% CI: 1.60–13.94, *P* = 0.005) after controlling for maternal education, feeding practices household income, and number of household members | Include (8/8) |
| Verawati et al., 2021^35^ | Kampar Regency, Riau | 55 toddlers (24-59 months) | Food Insecurity and Experience Scale  (FIES) | - 58.2% households were food insecure (32 of 55 households) | 52.7% (29 out of 55 children) were stunted | - There was a significant relationship between household food insecurity and stunting among under-five children (*P* = 0.001) | Include (6/8) |
| Qatrunnada et al., 2023^36^ | Banjar Regency, South Kalimantan | 88 Toddlers (24-59 Months) | Expenditure-based approach using household food expenditure ≥60% as indicator of food insecurity | - 84.1% households were classified as food insecure | 44.3% children were stunted | - There was no significant association between household food security and stunting (*P* = 0.292) | Include (7/8) |
| Aisyah et al., 2024^37^ | Tasikmalaya City, West Java | 142 toddlers aged 2–5 years (71 stunted, 71 non-stunted) | FIES | Stunted Children:   - Vulnerable: 23 (32.4%) - Moderate Vulnerable: 36 (50.7%) - Secure: 12 (16.9%)   Non-Stunted Children:   - Vulnerable: 16 (22.5%) - Moderate Vulnerable: 26 (36.6%) - Secure: 29 (40.8%) | 50.0% prevalence of stunting (by case definition) | - Significant association between household food insecurity and stunting in bivariate analysis (*P* = 0.007). - Multivariate analysis showed food insecurity (*P* = 0.002) as key predictors of stunting. Other factors: underweight, energy intake, carbohydrate, protein, and maternal nutrition knowledge | Include (8/8) |
| Rifayanto et al., 2022^38^ | Kepulauan Seribu Regency, Jakarta | 41 mothers with children aged 36–59 months | HFIAS | - Food secure: 19.5% - Mild food insecurity: 46.3% - Moderate food insecurity: 26.8% - Severe food insecurity: 7.3% | Stunting: 14.6% | - A significant moderate correlation was found between food security and stunting (r = 0.43, *P* = 0.005) | Include (6/8) |
| Fentiana et al., 2019^39^ | Langkat Regency, North Sumatra | 45 households with children aged 0–59 months | US-HFSSM– short version | - Food Insecure: 55.6% - Food Secure: 44.4% | 42.2% of children were stunted | - There is a statistically significant association between household food insecurity and stunting in children under five (*P* = 0.017). | Include (6/8) |
| Masthalina et al., 2021^40^ | Tapanuli Regency, North Sumatra | 59 children under five years and their households, most heads of households are fishermen (78%) | HFIAS | - 81.4% (48 of 59 households were food insecure) | Stunting: 35.6% | - Stunting: Not statistically significant (*P* = 0.297; OR = 2.95), but numerically higher in food-insecure families | Include (6/8) |
| Masitoh et al., 2022^41^ | Indonesia | 82,777 children under five years (0–59 months) from the 2021 Indonesian Nutritional Status Survey (INSS) | FIES | - Mild: 84.8% - Moderate: 11.9% - Severe: 3.3% | Stunting: 23.8% | - Bivariate analysis: Moderate food insecurity: PR = 1.24 (95% CI: 1.18–1.31) Severe food insecurity: PR = 1.39 (95% CI: 1.27–1.53) - Multivariate analysis (rural only): Moderate: aPR = 1.09 (95% CI: 1.02–1.16), *P* = 0.008 Severe: aPR = 1.15 (95% CI: 1.04–1.28), *P* = 0.009 - Adjusted for: mother’s education, household size, wealth index, low birth weight, infection history. - No significant association in urban areas. | Include (8/8) |
| Adhyanti et al., 2022^42^ | Palu City, Central Sulawesi | 96 households with children under five years (disaster survivors, post-earthquake) | HFIAS | - Food secure: 46.9% - Mild FI: 28.1% - Moderate FI: 15.6% - Severe FI: 9.4% | Stunted: 30.2% | Significant association between food security and stunting (*P* = 0.041) | Include (7/8) |
| Wijaya et al., 2023^43^ | Purworejo Regency, Central  Java Province | 250 households with children under five | Assessed using calorie adequacy, classified as: Food secure (≥2200 kcal/day per capita) Food insecure (<2200 kcal/day per capita) | - Not explicitly reported in prevalence % but analyzed using dummy variable (secure vs insecure) | Stunting prevalence in the district was 15.7% | Household food security was found to be a significant determinant of stunting with: OR = -0.679, *P* = 0.001 | Include (8/8) |
| Nashira et al., 2024^44^ | Urban Area Sungai Penuh  City, Jambi Province | 308 children aged 0–59 months living in urban areas Data source: 2022 Indonesian Nutrition Status Survey (INSS) | Categorized as “Poor”, “Borderline”, and “Acceptable” food security Derived from INSS 2022 dataset | - Acceptable food security: 97.8% - Borderline: 2.2% - Poor: 0.0% | Child stunting: 30.8% | - Bivariate: Food security not significantly associated with stunting (*P* = 0.385) - Multivariate: Food security still not significantly associated (OR = 2.33; 95% CI: 0.30–18.31; *P* = 0.421) | Include (8/8) |
| Sanggelorang et al., 2024^45^ | East Bolaang Mongondow Regency, North Sulawesi | 8,893 children under five years old (0–59 months) and their households, from the 2021 Indonesian Nutritional Status Survey (INSS) | FIES | 40.6% (3,607 out of 8,893 households were food vulnerable) | Total food vulnerable group: 3,607 children   - Stunted: 1,418 children (68.7%) - Not stunted: 2,189 children (32.0%)   Total food secure group: 5,286 children   - Stunted: 645 children (31.3%) - Not stunted: 4,641 children (68.0%) | There is a strong and significant association between food vulnerability and stunting among children under five (OR = 4.66 (95% CI: 4.19–5.18), *P* < 0.001) | Include (6/8) |

**Supplementary Table 2C. Association Between HFS and Underweight (Cross sectional Studies)**

| **Author** | **Location** | **Participants** | **HFI Tool** | **Prevalence of HFI** | **Prevalence of Outcome** | **Main result** | **JBI Quality** |
| --- | --- | --- | --- | --- | --- | --- | --- |
| Priawantiputri et al., 2021^29^ | Kolaka Timur Regecy, Southeast Sulawesi | 429 children aged <2 years | US-HFSSM– short version | - High or marginal food secure: 53.80% - Food insecure without hunger: 34.0% - Food insecure with hunger: 11.4% | Underweight: 15.9% | - No significant association between household food security and Underweight (*P* = 0.714) | Include 7/8) |
| Sutriningsih and Lastri., 2017^49^ | Malang Regency, East Java, | 96 families with toddlers aged 1–5 years | US-HFSSM | - High food security: 76% of households - Marginal: 3.1% - Low: 16.7% - Very low: 4.2% | - Underweight: 6 children (6.3%) - Severely underweight: 1 child (1.0%) | - There were a strong and significant relationship between household food security and nutritional status of toddlers in Malang post-Bromo eruption, with children from food secure households more likely to have good nutrition (*P* = 0.049, r = 1.00). | Include (7/8) |
| Riski et al., 2019^50^ | Surabaya City, East Java | 64 households with children aged 1–5 years | US-HFSSM | - 60.9% of households were classified as food insecure - Only 39.1% were food secure | - Severely underweight: 1. 6% - Underweight: 29.7% | - Household Food Security: Significantly correlated with underweight (*P* < 0.001), r = 0.46 | Include (6/8) |
| Rifayanto et al., 2022^38^ | Kepulauan Seribu Regency, Jakarta | 41 mothers with children aged 36–59 months | HFIAS | - Food secure: 19.5% - Mild food insecurity: 46.3% - Moderate food insecurity: 26.8% - Severe food insecurity: 7.3% | Underweight: 12.2% | - Household food security was significantly correlated with underweight in children (r = 0.50, *P* = 0.001) | Include (6/8) |
| Masthalina et al., 2021^40^ | Tapanuli Regency, North Sumatra | 59 children under five years, most heads of households are fishermen (78%) | HFIAS | 81.4% (48 of 59 households were food insecure) | Underweight: 50.8% | - Underweight: Food insecurity associated with increased risk of underweight (*P* = 0.039; OR = 6.30) | Include (7/8) |
| Adhyanti et al., 2022^42^ | Palu City, Central Sulawesi | 97 households with children under five (disaster survivors, post-earthquake) | HFIAS | - Food secure: 46.9%; - Mild FI: 28.1%; - Moderate FI: 15.6%; - Severe FI: 9.4% | Underweight: 18.7% | - No significant association with underweight (*P* = 0.202) | Include (8/8) |

**Supplementary Table 2D. Association Between HFS and Wasting (Cross sectional Studies)**

| **Author** | **Location** | **Participants** | **HFI Tool** | **Prevalence of HFI** | **Prevalence of Outcome** | **Main result** | **JBI Quality** |
| --- | --- | --- | --- | --- | --- | --- | --- |
| Priawantiputri et al., 2021^29^ | Kolaka Timur Regecy, Southeast Sulawesi | 429 children aged <2 years with their mothers | US-HFSSM– short version | - High or marginal food secure: 53.80% - Food insecure without hunger: 34.0% - Food insecure with hunger: 11.4% | Wasting: 8.4% | - No significant association between household food security and Wasting (*P* = 0.250) | Include (7/8) |
| Firmansyah et al., 2024^51^ | Indonesia | 4,391 children (1 to 5 years), using secondary data from the Indonesia Family Life Survey (IFLS) 2014 | Food Frequency Questionnaire (17 items) aggregated into Food Consumption Score (FCS); Based on World Food Programme (WFP) | - Food secure: 21.5% - Moderately food insecure: 26.3% - Severely food insecure: 52.1% | Severely wasted: 3.1% | - Moderate food insecurity increased the odds of severe wasting (OR: 1.81; 95% CI: 1.21–2.71; *P* < 0.050) - Severe food insecurity increased the odds of severe wasting (OR: 1.66; 95% CI: 1.069–2.570; *P* < 0.050) | Include (6/8) |
| Firmansyah et al., 2024^51^ | Indonesia | 4,391 children (1 to 5 years), using secondary data from the Indonesia Family Life Survey (IFLS) 2014 | Food Frequency Questionnaire (17 items) aggregated into FCS; WFP | - Food secure: 21.5% - Moderately food insecure: 26.3% - Severely food insecure: 52.1% | Wasted: 7.7% | - Moderate food insecurity increased the odds of wasting (OR: 2.02; 95% CI: 1.55–2.62; *P* < 0.001) - Severe food insecurity increased the odds of wasting (OR: 1.87; 95% CI: 1.40–2.48; *P* < 0.001) | Include (6/8) |
| Sihotang and Rumida; 2020^52^ | Deli Serdang Regency, North Sumatra | 78 toddlers (13–59 months) | Food security was categorized as follows:   - Food secure: if food expenditure was low (<60%) and energy intake was adequate (>80% of RDA). - Vulnerable to food insecurity: if food expenditure was high (≥60%) and energy intake was adequate (>80% of RDA). - Food insufficient: if food expenditure was low (<60%) and energy intake was inadequate (≤80% of RDA). - Food insecure: if food expenditure was high (≥60%) and energy intake was inadequate (≤80% of RDA). | - Food insecure: 41 households (52.6%) Food insufficient: 10 (12.8%) Vulnerable to food insecurity: 17 (21.8%) Food secure: 10 (12.8%) | - Severely wasting: 5 children (6.4%) - Wasting: 17 children (21.8%) | - No significant association between household food security and nutritional status of toddlers (*P* = 0.488) | Include (7/8) |
| Rifayanto, et al., 2022^38^ | Kepulauan Seribu Regency, Jakarta | 41 mothers with children aged 36–59 months | HFIAS | - Food secure: 19.5% - Mild food insecurity: 46.3% - Moderate food insecurity: 26.8% - Severe food insecurity: 7.3% | Wasting: 12.2% | - No significant correlation was observed between household food security and wasting (r = 0.05, *P* = 0.770) | Include (6/8) |
| Masthalina et al., 2021^40^ | Tapanuli Regency, North Sumatra | 59 children under five years and their households, most heads of households are fishermen (78%) | HFIAS | - 81.4% (48 of 59 households were food insecure) | Wasting: 10.2% | - Not statistically significant (*P* = 0.581; OR = 1.14), rare overall | Include (6/8) |
| Adhyanti et al., 2022^42^ | Palu City, Central Sulawesi | 98 households with children under five (disaster survivors, post-earthquake) | HFIAS | - Food secure: 46.9%; - Mild FI: 28.1%; - Moderate FI: 15.6%; - Severe FI: 9.4% | Wasted: 15.6% | - No significant association with wasting (*P* = 0.253) | Include (7/8) |

**Supplementary Table 2E. Association Between HFS and Overnutrition (Cross sectional Studies)**

| **Author** | **Location** | **Participants** | **HFI Tool** | **Prevalence of HFI** | **Prevalence of Outcome** | **Main result** | **JBI Quality** |
| --- | --- | --- | --- | --- | --- | --- | --- |
| Hidayati, 2023^48^ | Pasuruan Regency, East Java | 100 children under five years (6–59 months) | US-HFSSM | - High food security: 42.0% - Marginal food security: 47.0% - Low food security: 9.0% - Very low food security: 2.0% | - Possible risk of overweight: 13.0% - Overweight: 11.0% - Obese: 4.0% | - There was a statistically significant association between household food security status and W/H classification (*P* = 0.042) | Include (7/8) |
| Firmansyah et al., 2024^51^ | Indonesia | 4,391 children (1 to 5 years), using secondary data from the Indonesia Family Life Survey (IFLS) 2014 | Food Frequency Questionnaire (17 items) aggregated into Food FCS; WFP | - Food secure: 21.5% - Moderately food insecure: 26.3% - Severely food insecure: 52.1% | Possible risk of overweight: 11.1% | - Moderately food insecure children had a 1.45 times higher risk of possible overweight than food secure children. OR = 1.45; 95% CI: 1.149–1.83; *P* < 0.050 - Severely food insecure children had a 1.82 times higher risk of possible overweight. OR = 1.82; 95% CI: 1.44–2.30; *P* < 0.001 | Include (6/8) |
| Firmansyah et al., 2024^51^ | Indonesia | 4,391 children (1 to 5 years), using secondary data from the Indonesia Family Life Survey (IFLS) 2014 | Food Frequency Questionnaire (17 items) aggregated into Food FCS; WFP | - Food secure: 21.5% - Moderately food insecure: 26.3% - Severely food insecure: 52.1% | Overweight: 4.8% | - Moderately food insecure children had a 1.59 times higher odds of being overweight. OR = 1.59; 95% CI: 1.15–1.83; *P* < 0.05 - Severely food insecure children had a 1.461 times higher odds of overweight. OR = 1.46; 95% CI: 1.02–2.09; *P* < 0.050 | Include (6/8) |
| Firmansyah et al., 2024^51^ | Indonesia | 4,391 children (1 to 5 years), using secondary data from the Indonesia Family Life Survey (IFLS) 2014 | Food Frequency Questionnaire (17 items) aggregated into Food FCS; WFP | - Food secure: 21.5% - Moderately food insecure: 26.3% - Severely food insecure: 52.1% | Obese: 4.2% | - Moderately food insecure children had a 2.22 times higher odds of obesity. OR = 2.22; 95% CI: 1.58–3.11; *P* < 0.050 - Severely food insecure children had a 1.59 times higher odds of obesity. OR = 1.59; 95% CI: 1.07–2.35; *P* < 0.050 | Include (6/8) |
| Sihotang and Rumida; 2020^52^ | Deli Serdang Regency, North Sumatra | 78 toddlers (13–59 months) | Food security was categorized as follows:   - Food secure: if food expenditure was low (<60%) and energy intake was adequate (>80% of RDA). - Vulnerable to food insecurity: if food expenditure was high (≥60%) and energy intake was adequate (>80% of RDA). - Food insufficient: if food expenditure was low (<60%) and energy intake was inadequate (≤80% of RDA). - Food insecure: if food expenditure was high (≥60%) and energy intake was inadequate (≤80% of RDA). | - Food insecure: 41 households (52.6%) - Food insufficient: 10 (12.8%) - Vulnerable to food insecurity: 17 (21.8%) - Food secure: 10 (12.8%) | Overweight: 10 children (12.8%) | - No significant association between household food security and nutritional status of toddlers (*P* = 0.488) | Include (7/8) |

**Supplementary Table 2F. Association Between HFS and Anaemia (Cross sectional Studies)**

| **Author** | **Location** | **Participants** | **HFI Tool** | **Prevalence of HFI** | **Prevalence of Outcome** | **Main result** | **JBI Quality** |
| --- | --- | --- | --- | --- | --- | --- | --- |
| Rohmah et al., 2024^53^ | Karawang Regency, West Java | 100 children (6 - 23 months) | HFIAS | - Food Secure: 76.0% - Food Insecure: 24.0% | Anaemia: 25.0% | - Bivariate analysis: - Food insecurity significantly associated with anaemia (OR = 4.85; 95% CI: 1.79–13.15; *P* = 0.003) - Multivariate analysis: - Food insecurity remained the strongest predictor (AOR = 10.05; *P* = 0.003) - Controlled variables in multivariate model:  1. Maternal age 2. Maternal education 3. Maternal employment status 4. Knowledge about anaemia 5. Parenting practic | Include (8/8) |
| Priawantiputri et al., 2021^29^ | Kolaka Timur Regecy, Southeast Sulawesi | 429 children aged <2 years with their mothers | US-HFSSM– short version | - High or marginal food secure: 53.80% - Food insecure without hunger: 34.0% - Food insecure with hunger: 11.4% | Anaemia: 63.6% | Significant association between food insecurity with hunger and anaemia (*P* = 0.037) | Include (7/8) |
