# Supplementary material for: The role of household food insecurity in malnutrition among Indonesian children under 5 years of age: a systematic review and meta-analysis (2015–2025)
Source: Public Health Nutr. 2026 Mar 26;29(1):e86. doi: 10.1017/S1368980026102365 (PMC13112310; doi:10.1017/S1368980026102365)
Supplement: Sutrisno et al. supplementary material 2 — Sutrisno et al. supplementary material [file S1368980026102365sup002.docx]

**Supplementary Table 3. GRADE evidence profile for meta-analysis.**

| **Outcome** | **Risk of Bias** | **Inconsistency** | **Indirectness** | **Imprecision** | **Publication Bias** | **GRADE Rating** |
| --- | --- | --- | --- | --- | --- | --- |
| Stunting (Cross-sectional) | No serious limitations; most studies used validated HFI tools and clear definitions of outcomes. | None; I² = 0.0%, highly consistent across studies. | None; directly measured HFI and stunting in target population. | None; narrow CIs and large sample sizes. | Low concern; symmetrical funnel plot and sufficient study number. | Moderate to High |
| Stunting (Case-control) | No serious limitations; consistent case-control methodology and adjustment for confounders. | None; I² = 0.0%, effect estimates closely aligned. | None; case-control designs matched for context and outcome. | None; precise estimates with tight CIs. | Low concern; no sign of small-study effects. | Moderate to High |
| Wasting | Moderate; some studies had small sample sizes or unclear outcome classification. | Serious; I² = 76.0%, moderate to high variability in effect size. | None; studies conducted in relevant populations using standard definitions. | Serious; wide CIs and imbalanced weights between studies. | Low concern; unlikely influence despite heterogeneity. | Moderate |
| Underweight | Moderate; several studies had wide CIs or lacked adjustment for covariates. | Serious; I² = 83.0%, variation in magnitude and direction of effect. | None; outcomes measured using BB/U, relevant to target group. | Serious; broad CIs in multiple studies. | Low concern; consistent direction of effect. | Moderate |
| Overnutrition | Moderate; limited number of studies and some relied on self-reported outcomes. | None; I² = 0.0%, although only two studies included. | None; overnutrition defined via BB/TB, appropriate for young children. | Some concerns; limited study number and minor CI overlap with null. | Low concern; although study number is small, effects are consistent. | Moderate |
| Anaemia | No serious limitations; both studies used valid tools and direct outcome measures. | Moderate; I² = 52.0%, some variation across studies. | None; anaemia measured biomedically in children under five. | None; both studies showed strong effects and narrow CIs. | Low concern; both studies were peer-reviewed and from reputable sources. | Moderate to High |
