# Supplementary material for: The role of household food insecurity in malnutrition among Indonesian children under 5 years of age: a systematic review and meta-analysis (2015–2025)
Source: Public Health Nutr. 2026 Mar 26;29(1):e86. doi: 10.1017/S1368980026102365 (PMC13112310; doi:10.1017/S1368980026102365)
Supplement: Sutrisno et al. supplementary material 3 — Sutrisno et al. supplementary material [file S1368980026102365sup003.docx]

**Supplementary Table 1A. JBI Analysis for Case-Control Studies**

| No | Author, Year | Were groups comparable other than the disease presence? | Were cases and controls matched appropriately? | Were same criteria used to identify cases and controls? | Was exposure measured in a valid and reliable way? | Was exposure measured the same way for both groups? | Were confounding factors identified? | Were strategies to deal with confounding stated? | Were outcomes assessed in a standard, valid, and reliable way for both groups? | Was the exposure period long enough to be meaningful? | Was appropriate statistical analysis used? | Total 'Yes' | Decision |
| --- | --- | --- | --- | --- | --- | --- | --- | --- | --- | --- | --- | --- | --- |
| 1 | Adelina et al., 2018^26^ | Yes | Yes | Yes | Yes | Yes | No | No | Yes | Unclear | Yes | 7 | Include |
| 2 | Fadzila and Tertiyus 2019^22^ | Yes | Yes | Yes | Yes | Yes | Unclear | No | Yes | Yes | Yes | 8 | Include |
| 3 | Raharja et al., 2019^27^ | Yes | Yes | Yes | Yes | Yes | Yes | Yes | Yes | Unclear | Yes | 9 | Include |
| 4 | Wado et al., 2019^24^ | Yes | Yes | Yes | Yes | Yes | No | No | Yes | Unclear | Yes | 7 | Include |
| 5 | Rohmawati et al., 2023^28^ | Yes | Yes | Yes | Yes | Yes | No | No | Yes | Yes | Yes | 8 | Include |
| 6 | Wardani et al., 2023^25^ | Yes | Yes | Yes | Yes | Yes | No | No | Yes | Yes | Yes | 8 | Include |
| 7 | Frisnoiry et al., 2024^23^ | Yes | Yes | Yes | Yes | Yes | Yes | Yes | Yes | Unclear | Yes | 9 | Include |

**Supplementary Table 1B. JBI Analysis for Cross-Sectional Studies**

| No | Author, Year | 1. Were the inclusion criteria clearly defined? | 2. Were the study subjects and setting described in detail? | 3. Was the exposure measured in a valid and reliable way? | 4. Were objective, standard criteria used for outcome measurement? | 5. Were confounding factors identified? | 6. Were strategies to deal with confounding factors stated? | 7. Were outcomes measured in a valid and reliable way? | 8. Was appropriate statistical analysis used? | Total 'Yes' | Decision |
| --- | --- | --- | --- | --- | --- | --- | --- | --- | --- | --- | --- |
| 1 | Adhyanti et al., 2022^42^ | Yes | Yes | Yes | Yes | Yes | No | Yes | Yes | 7 | Include |
| 2 | Aisyah et al., 2024^37^ | Yes | Yes | Yes | Yes | Yes | Yes | Yes | Yes | 8 | Include |
| 3 | Asparian et al., 2020^34^ | Yes | Yes | Yes | Yes | Yes | Yes | Yes | Yes | 8 | Include |
| 4 | Fentiana et al., 2019^39^ | Yes | Yes | Yes | Yes | Unclear | No | Yes | Yes | 6 | Include |
| 5 | Firmansyah et al., 2024^51^ | Yes | Yes | Yes | Yes | No | No | Yes | Yes | 6 | Include |
| 6 | Gunawan and Septriana, 2019^46^ | Yes | Yes | Yes | Yes | No | No | Yes | Yes | 6 | Include |
| 7 | Hidayati, 2023^48^ | Yes | Yes | Yes | Yes | Yes | No | Yes | Yes | 7 | Include |
| 8 | Islamiah et al., 2022^47^ | Yes | Yes | Yes | Yes | Yes | No | Yes | Yes | 7 | Include |
| 9 | Mahmudiono et al., 2018^32^ | Yes | Yes | Yes | Yes | Yes | Yes | Yes | Yes | 8 | Include |
| 10 | Masitoh et al., 2022^41^ | Yes | Yes | Yes | Yes | Yes | Yes | Yes | Yes | 8 | Include |
| 11 | Masthalina et al., 2021^40^ | Yes | Yes | Yes | Yes | No | No | Yes | Yes | 6 | Include |
| 12 | Nashira et al., 2024^44^ | Yes | Yes | Yes | Yes | Yes | Yes | Yes | Yes | 8 | Include |
| 13 | Priawantiputri, 2021^29^ | Yes | Yes | Yes | Yes | Yes | No | Yes | Yes | 7 | Include |
| 14 | Qatrunnada et al., 2023^36^ | Yes | Yes | Yes | Yes | Yes | No | Yes | Yes | 7 | Include |
| 15 | Rifayanto, 2019^38^ | Yes | Yes | Yes | Yes | No | No | Yes | Yes | 6 | Include |
| 16 | Riski et al., 2019^50^ | Yes | Yes | Yes | Yes | No | No | Yes | Yes | 6 | Include |
| 17 | Rohmah et al., 2024^53^ | Yes | Yes | Yes | Yes | Yes | Yes | Yes | Yes | 8 | Include |
| 18 | Safitri and Nindya, 2017^31^ | Yes | Yes | Yes | Yes | Yes | No | Yes | Yes | 7 | Include |
| 19 | Sanggelorang et al., 2024^45^ | Yes | Yes | Yes | Yes | Unclear | No | Yes | Yes | 6 | Include |
| 20 | Sihotang and Rumida, 2020^52^ | Yes | Yes | Yes | Yes | Yes | No | Yes | Yes | 7 | Include |
| 21 | Sutriningsih, 2017^49^ | Yes | Yes | Yes | Yes | Yes | No | Yes | Yes | 7 | Include |
| 22 | Utami et al., 2015^30^ | Yes | Yes | Yes | Yes | Yes | Yes | Yes | Yes | 8 | Include |
| 23 | Verawati et al., 2021^35^ | Yes | Yes | Yes | Yes | No | No | Yes | Yes | 6 | Include |
| 24 | Widyaningsih et al., 2019^33^ | Yes | Yes | Yes | Yes | Yes | Yes | Yes | Yes | 8 | Include |
| 25 | Wijaya et al., 2023^43^ | Yes | Yes | Yes | Yes | Yes | Yes | Yes | Yes | 8 | Include |
